# A successful transfemoral transcatheter aortic valve replacement case with VIABAHN® VBX balloon‐expandable stent‐graft and long Dryseal sheath for challenging access route

**DOI:** 10.1002/ccr3.8178

**Published:** 2023-11-13

**Authors:** Tomohiro Kawaguchi, Kosuke Seiyama, Satoko Ugawa, Kazumasa Nosaka, Masayuki Doi

**Affiliations:** ^1^ Department of Cardiology Kagawa Prefectural Central Hospital Takamatsu Japan

**Keywords:** endovascular therapy, shaggy aorta, transcatheter aortic valve replacement

## Abstract

**Key Clinical Message:**

A combination of long large‐bore sheath and balloon‐expandable stent‐graft can be effective to challenging access route in transfemoral transcatheter aortic valve replacement.

**Abstract:**

An 88‐year‐old female with symptomatic severe aortic stenosis underwent transcatheter aortic valve replacement (TAVR). Multidetector computed tomography demonstrated a small aortic annulus, shaggy aorta, and significant stenosis with heavily calcified atherosclerotic lesions in the bilateral common iliac arteries (CIAs). TAVR with Evolut™ Pro+ via alternative approach was considered; however, our heart team concluded that the patient was unsuitable for the procedure due to anatomical reasons, patient frailty, and medication history. Finally, transfemoral TAVR with endovascular therapy (EVT) and 18Fr‐65 cm‐Dryseal was adopted for the site. Following EVT with VIABAHN® VBX balloon‐expandable stent‐graft (VBX) implantation to the right ostial CIA lesion, 18Fr‐65 cm‐Dryseal was advanced to the ascending aorta through VBX, and Evolut™ Pro+26 mm was successfully implanted without any complication. At the 2‐month follow‐up, the patient reported a significant improvement in shortness of breath and did not present any evidence of atheroembolism. Transfemoral TAVR with 18Fr‐65 cm‐Dryseal to shaggy aorta can be feasible depending on the plaque distribution, and VBX implantation to a heavily calcified ostial CIA lesion was safe and effective for obtaining enough lumen for a large‐bore sheath.

## Graphical abstract

Challenging access route for transfemoral approach.

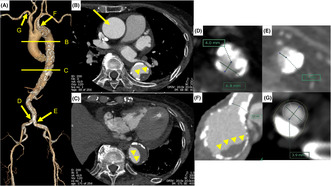

## 1 INTRODUCTION

Transcatheter aortic valve replacement (TAVR) is an established treatment option for patients with severe aortic stenosis (AS). The transfemoral approach is the first‐line option for the approach site, and majority of TAVR procedures are performed via this site.^1^ Alternative approach sites are considered for patients with minimum iliofemoral lumen diameter less than 5 mm, severe tortuosity or calcification, chronic arterial dissection/thrombus, morbid obesity, or severe abdominal aortic atherosclerosis.^2^ However, we have encountered patients who were anatomically suitable but were not appropriate for relatively invasive alternative approaches; transthoracic approaches, due to concurrent and long‐term steroid or immunosuppressant administration, advanced age, and/or clinical frailty. In those situations, we have no choice but to perform transfemoral TAVR. This is first report of a successful transfemoral TAVR case with a shaggy aorta and heavily calcified stenosis in the bilateral common iliac arteries (CIAs), which was generally considered unsuitable for transfemoral TAVR, using a combination of endovascular therapy (EVT) with a stent‐graft and advancing a large‐bore long sheath to bypass a shaggy aorta.

## 2 CASE REPORT

An 88‐year‐old female with hypertension, dyslipidemia, and rheumatoid arthritis was referred to our hospital with complaints of progressive shortness of breath. On physical examination, the patient showed a 3/6 systolic murmur at the right lower sternal border. Electrocardiogram showed sinus rhythm and no atrioventricular block or bundle branch block. Laboratory investigations indicated elevated white blood cell of 10,700/μL, C‐reactive protein of 0.96 mg/dL, stage 3b chronic kidney disease (estimated glomerular filtration rate: 39.2 mL/min/1.73 m^2^), and elevated brain natriuretic peptide level of 193 pg/mL. Transthoracic echocardiography revealed normal left ventricular systolic function and a calcified tricuspid aortic valve with aortic valve area of 0.43 cm^2^, peak velocity of 4.3 m/s, and mean pressure gradient of 39 mmHg. No other significant valvular disease was detected. The patient was diagnosed with symptomatic severe AS and was concluded by our heart team to be a candidate for TAVR due to advanced age and clinical frailty scale of 5.

**FIGURE 1:**
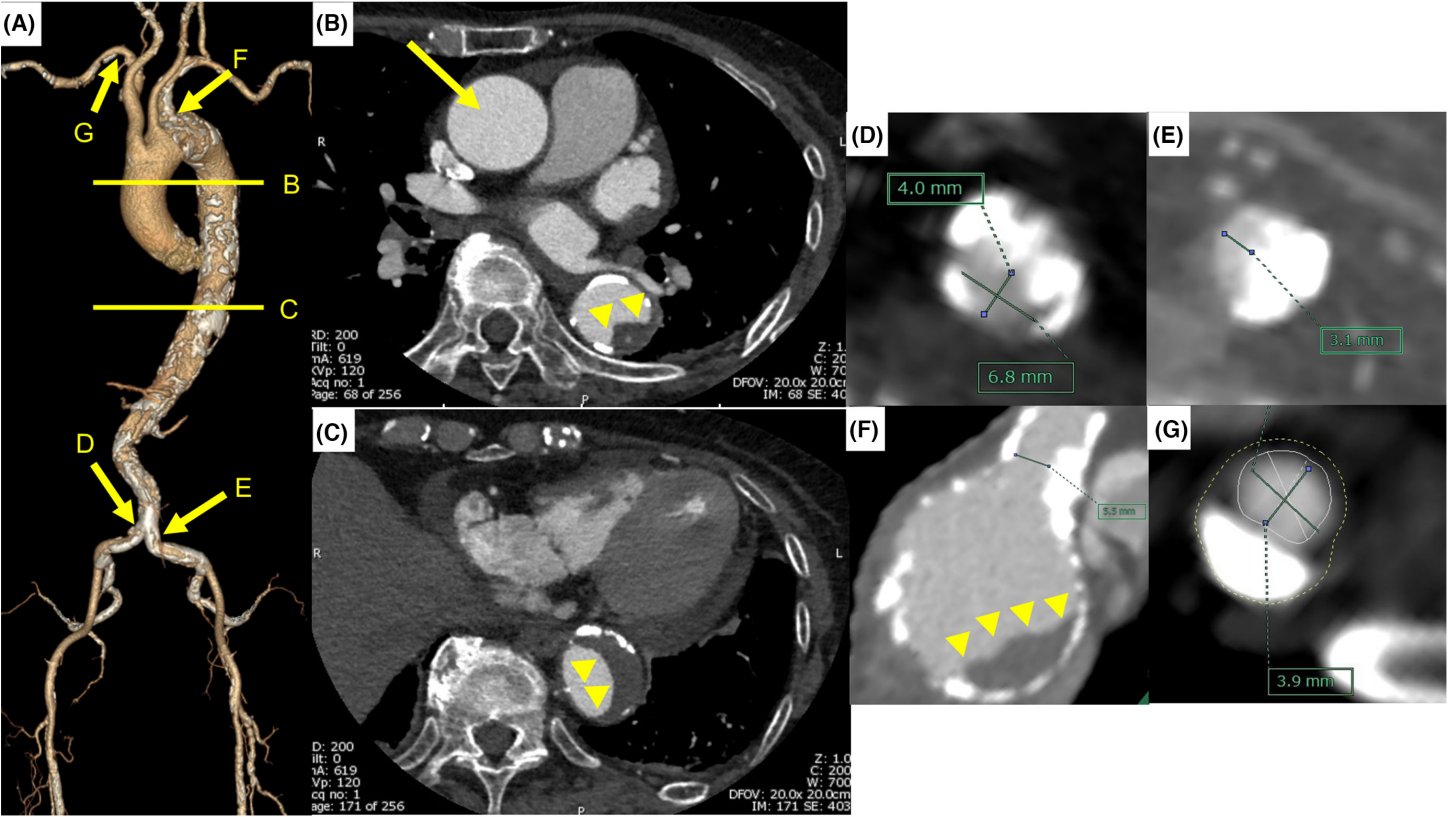
Preprocedural CT images. (A) A volume rendering image. Line B and C indicate the sliced level of CT scan and arrows D–G indicate the location of the cross‐sectional CT images. (B) An axial CT image of the ascending and thoracic descending aorta. An arrow indicates the ascending aorta. Arrowheads indicate the plaque in the descending aorta. (C) Another axial CT image of thoracic descending aorta. Arrowheads indicate the plaque in the descending aorta. (D) A cross‐sectional CT image of the right ostial CIA, minimal lumen diameter of 4.0 mm with heavy calcification. (E) A cross‐sectional CT image of the left ostial CIA, minimal lumen diameter of 3.1 mm with heavy calcification. (F) A cross‐sectional CT image of the aortic arch where the left subclavian artery branches, minimal lumen diameter of 5.5 mm with heavy calcification. Arrowheads indicate the plaque. (G) A cross‐sectional CT image of the right subclavian artery, minimal lumen diameter of 3.9 mm with eccentric calcification. CIA, common iliac artery; CT, computed tomography.

Multidetector computed tomography (MDCT) demonstrated an aortic annulus area of 300 mm^2^ and a perimeter of 64.4 mm. In those days, we were able to use two types of TAVs, which were Evolut™ Pro+ (Medtronic Inc., Minneapolis, MN, USA) and SAPIEN3™ (Edwards Life Sciences, Irvine, CA). Evolut™ Pro+, which has a self‐expandable and supra‐annular design, was reported to show better hemodynamic profile of TAV and less frequency of patient‐prosthesis mismatch for the small aortic annulus, compared with SAPIEN3™, which has a balloon‐expandable and intra‐annular design.^3^ Upon examination of the access route for the transfemoral approach, MDCT revealed a shaggy aorta, in which the plaque/thrombus was located in the aortic arch, from the descending thoracic aorta to the abdominal aorta (Figure 1A–C, Video S1), and significant stenosis with heavily calcified atherosclerotic lesions in the bilateral CIAs (Figure 1A, D, E). There were no anatomical difficulties in the right external iliac arteries or femoral puncture site. Although alternative approaches were considered, the approach through the left subclavian artery was deemed high risk for vascular injury due to a calcified ostial stenosis (Figure 1A, F), and the right subclavian approach was not suitable because the aortic angle was >30° and the minimal lumen diameter in right subclavian artery was 3.9 mm with eccentric calcification (Figure 1A, Figure 1F). Transaortic approach was anatomically possible; however, surgical intervention was not preferable because of the patient's frailty and the concomitant steroid (prednisolone of 10 mg daily) and immunosuppressant (salazosulfapyridine of 1000 mg daily) administration for rheumatoid arthritis. Transapical approach was also anatomically possible; however, Evolut™ Pro+ is impossible to use via transapical approach and neither is SAPIEN3™ because the aortic annular area of 300 mm^2^ was the range for 20 mm‐ SAPIEN3™, which is not commercially available other than transfemoral approach.

Our heart team concurred to perform right‐transfemoral TAVR. Advancing an 18Fr‐65 cm‐Dryseal (W. L. Gore & Associates, Flagstaff, AZ, USA) to the ascending aorta to minimize the risk of atheroembolism was thought to be possible because the plaques in the aortic arch were located in the aortic arch with lesser curvature (Figure 1F), and the surface of the plaque/thrombus from the descending thoracic aorta to the abdominal aorta was smooth (Video S1). Prior to the TAVR, EVT for the right CIA was scheduled to accommodate the 18Fr‐Dryseal.

**FIGURE 2:**
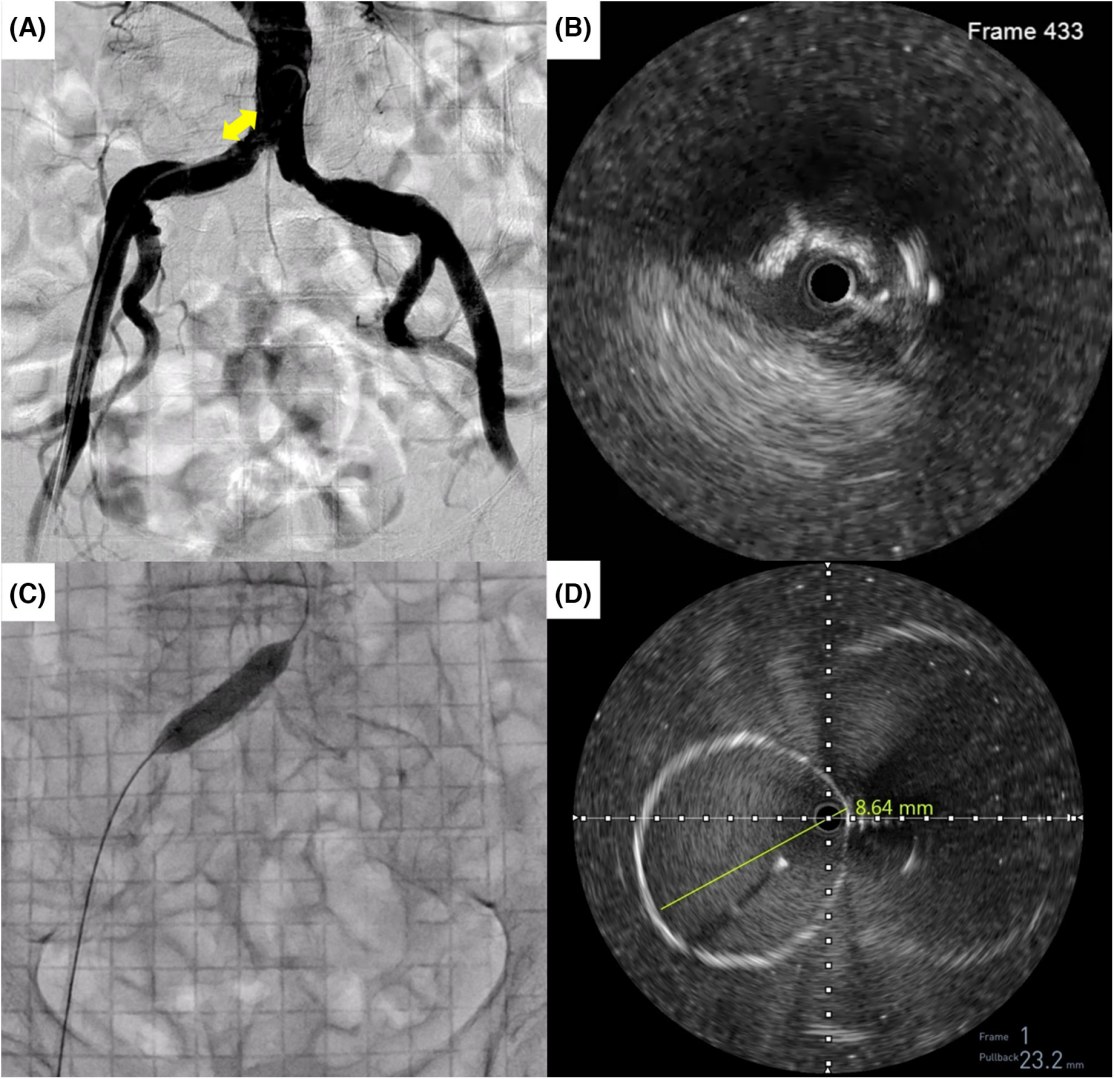
Fluoroscopic and IVUS images during EVT procedure. (A) Aortography. Bidirectional arrow indicates the translucency, which suggests calcification. (B) A cross‐sectional IVUS image of the right ostial CIA. (C) A 10 mm × 29 mm VIABAHN® VBX balloon‐expandable stent‐graft implantation. (D) A cross‐sectional IVUS image of minimal stent lumen diameter. CIA, common iliac artery; EVT, endovascular therapy; IVUS, intravascular ultrasound.

In the EVT procedure, an 8‐Fr sheath was inserted from the right common femoral artery and a 0.014‐inch wire was crossed to the ostial right CIA lesion. Intravascular ultrasound (IVUS) revealed a calcified nodule with eccentric distribution in the lesion (Figure 2A, B). Following the a 0.014‐inch wire exchange to a 0.035‐inch stiff wire, a 10 mm × 29 mm VIABAHN® VBX balloon‐expandable stent‐graft (VBX) (W. L. Gore & Associates, Flagstaff, AZ, USA) was directly implanted into the right CIA (Figure 2C). Through IVUS following VBX implantation, the minimal stent lumen diameter was 8.6 mm (Figure 2D), and an 18Fr‐Dryseal was considered to be accommodated.

**FIGURE 3:**
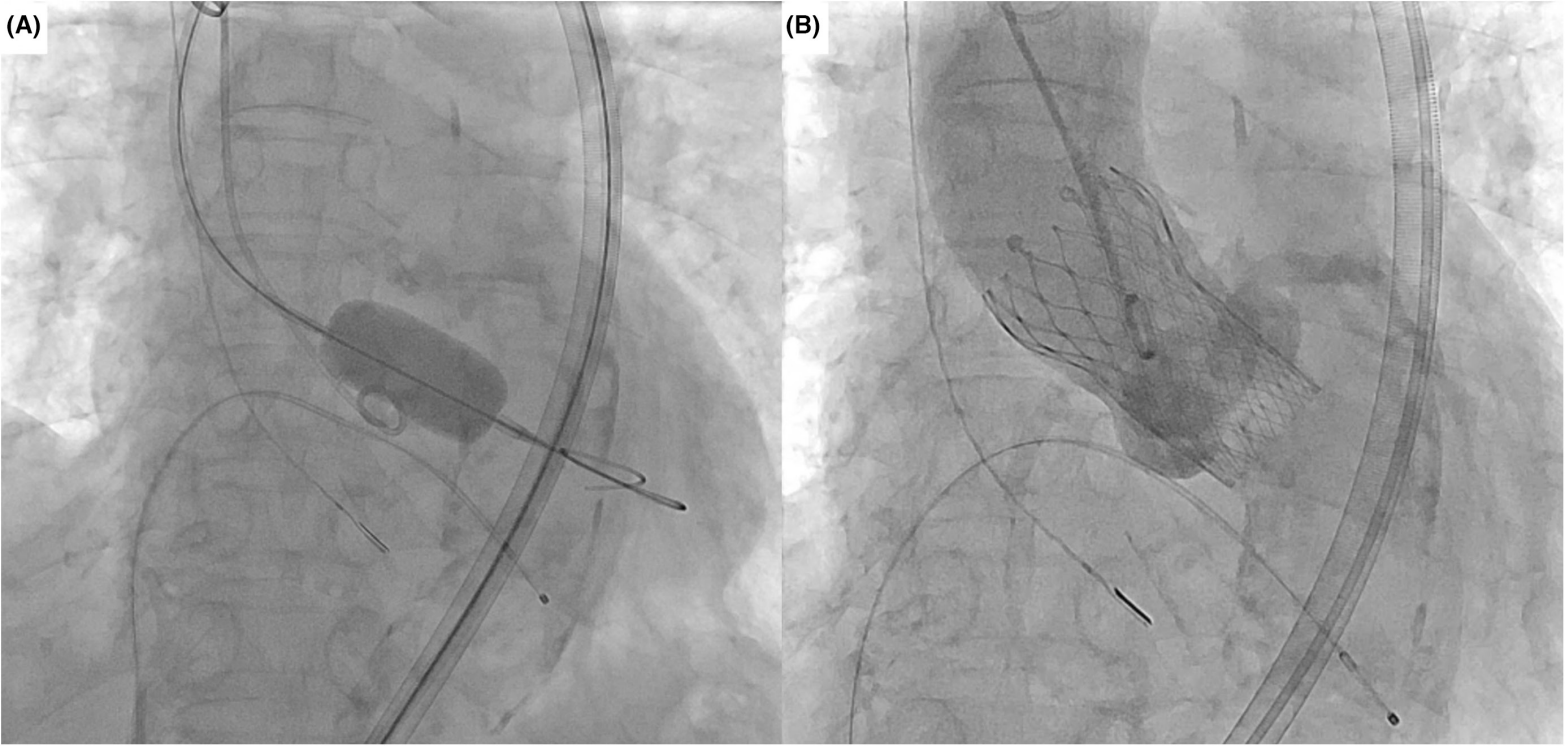
Fluoroscopic images during transcatheter aortic valve replacement procedure. (A) Predilatation with 18 mm‐retrograde Inoue balloon. (B) Aortography following 26‐mm Evolut™ Pro+ implantation.

TAVR was performed under general anesthesia and intracardiac echo guidance 4 weeks after the EVT procedure. Although the 18Fr‐65 cm‐Dryseal passed through the VBX with some degree of resistance, the Dryseal smoothly advanced to the ascending aorta using a Lunderquist® extra‐stiff wire (COOK Inc., Bloomington, IN, USA) after passing through VBX (Video S2). Following predilatation with an 18 mm‐retrograde Inoue balloon (Toray, Tokyo, Japan) (Figure 3A), a 26‐mm Evolut™ Pro+ was successfully implanted (Figure 3B). Four days after the TAVR, the patient was discharged without any complications.

At the 2‐month follow‐up, the patient reported a significant improvement in shortness of breath and did not present any evidence of atheroembolism. Transthoracic echocardiography demonstrated an effective orifice area of 1.48 cm^2^, an aortic peak velocity of 1.4 m/s, and a mean pressure gradient of 4 mmHg.

## 3 DISCUSSION

We describe a transfemoral TAVR case with heavily calcified stenosis in the CIA and the shaggy aorta, successfully treated with a combination of VIABAHN® VBX and 18Fr‐65 cm‐Dryseal. When performing TAVR, 65 cm‐Dryseal was reported to be effective for a patient with tortuous and shaggy aorta.^4^ Covered stent is an option for management of iliofemoral arterial rupture.^5^ Kawashima et.al reported successful transfemoral TAVR through the aortic stent‐graft after endovascular aortic repair of abdominal aortic aneurysm.^6^ However, to the best of our knowledge, this is the first reported case of simultaneous use of 18Fr‐65 cm‐Dryseal and VIABAHN® VBX for ensuring iliac access route for TAVR.

Peripheral arterial disease (PAD) is one of the challenging conditions for transfemoral TAVR. According to a large‐scale registry, of 19,960 patients undergoing transfemoral TAVR, 4810 (24.5%) had significant PAD.^7^ PAD is strongly correlated with an alternative approach to TAVR, which has a strong association with poorer clinical outcomes.^8^, ^9^ Two difficulties were encountered during the transfemoral TAVR procedure for this patient: the shaggy aorta and significant stenosis with heavily calcified stenosis in the bilateral CIA. Intra‐arterial procedures on the shaggy aorta can cause atheroembolism to multiple organs, including the brain, muscles, skin, eyes, kidneys, and gastrointestinal tract.^10^ The mortality rate of patients diagnosed with atheroembolism has been reported to be very high, up to 90%.^11^ Thus, avoiding atheroembolism was essential and bypassing the shaggy aorta with a 18Fr‐65 cm‐Dryseal was thought to be effective because it can minimize the number of passing through shaggy aortic sites. In this case, the surface of the plaque/thrombus from the descending thoracic aorta to the abdominal aorta was smooth and plaque distribution in the aortic arch was a lesser curvature. These anatomical features enabled us to determine transfemoral approach. Although the patient presented no evidence of atheroembolism in the 2‐month follow‐up, there is a possibility for chronic renal impairment, a progressive kidney dysfunction that is difficult to diagnose and is frequently underdiagnosed because it is clinically silent and extrarenal manifestations are usually absent.^12^ Frequent follow‐up or maintenance of close contact with the referred physician is important to detect whether the patient presents with manifestations of atheroembolism.

EVT for the right CIA was required to accommodate the 18Fr‐Dryseal, which has an outer diameter of 6.7 mm. A heavily calcified stenosis in the ostial right CIA was observed on preprocedural CT analysis, and the lesion was considered to be at high risk of vascular rupture because of its eccentric distribution on IVUS findings. According to the expert consensus, balloon‐expandable stents are better options for heavily calcified lesions or lesions with greater recoil than self‐expandable stents because more radial strength may be needed.^13^ The use of stent grafts instead of bare metal stents is recommended for cases involving heavily calcified atherosclerotic lesions, in which there is high potential for vascular rupture.^14^ The minimal stent lumen diameter may be necessary 1.0 mm over the outer diameter of Dryseal as a spatial margin inside the stent. IVUS measurement after stent implantation is also essential to ensure sufficient stent lumen diameter for accommodating a large‐bore sheath.

Two important methods were utilized for this patient; VBX implantation into the heavily calcified right CIA and advancing 18Fr‐65 cm‐Dryseal to the ascending aorta. The advantage of these methods was fully percutaneous and the least invasive procedures for the patient. The disadvantage of them was that there is no guarantee that atheroembolism can be avoided. However, meticulous CT analysis, understanding plaque distribution and condition, can help achieve successful TAVR procedure without any complication. Anatomically, transaortic approach was possible and it would be the standard procedure for the patient if the patient was suitable for the surgical intervention.

## 4 CONCLUSION

This case demonstrated the safety and effectiveness of pre‐TAVR‐procedural VBX implantation to a heavily calcified stenosis in the CIA for obtaining enough lumen diameter to accommodate a large‐bore sheath through the IVUS measurement. Moreover, the 18Fr‐65 cm‐Dryseal can be feasible depending on the plaque distribution in selected patients with shaggy aorta to minimize the risk of atheroembolism. Each method is independently feasible for the challenging access route in transfemoral TAVR.

## AUTHOR CONTRIBUTIONS

**Tomohiro Kawaguchi:** Conceptualization; data curation; investigation; methodology; project administration; writing – original draft. **Masayuki Doi:** Supervision. **Kosuke Seiyama:** Investigation. **Satoko Ugawa:** Investigation. **Kazumasa Nosaka:** Investigation.

## FUNDING INFORMATION

None.

## CONFLICT OF INTEREST STATEMENT

Tomohiro Kawaguchi is a clinical proctor for Medtronic Evolut transcatheter aortic valve. The other authors have no conflicts of interest to declare.

## CONSENT

Written informed consent was obtained from the patient for publication of this case report, including accompanying images.

## Supporting information


Videos S1–S2

## Data Availability

Data available on request from the authors.
