# Supplementary material for: A successful transfemoral transcatheter aortic valve replacement case with VIABAHN® VBX balloon‐expandable stent‐graft and long Dryseal sheath for challenging access route
Source: Clin Case Rep. 2023 Nov 13;11(11):e8178. doi: 10.1002/ccr3.8178 (PMC10643312; doi:10.1002/ccr3.8178)
Supplement: Supplementary file 1 — Videos S1–S2 [file CCR3-11-e8178-s001.zip › Video S1-S2 captions.docx]

Video S1. Continuous axial images of computed tomography.

Video S2 The 18Fr-65 cm-dryseal insertion from right femoral artery to the ascending aorta.
